# The complex tibial organ of the New Zealand ground weta: sensory adaptations for vibrational signal detection

**DOI:** 10.1038/s41598-017-02132-1

**Published:** 2017-05-17

**Authors:** Johannes Strauß, Kathryn Lomas, Laurence H. Field

**Affiliations:** 1 Justus-Liebig-Universität Gießen, Institute for Animal Physiology, AG Integrative Sensory Physiology, Gießen, Germany; 2 CSIRO Manufacturing Business Unit, Clayton, Victoria 3168 Australia; 3 University of Canterbury, School of Biological Sciences, Christchurch, New Zealand

## Abstract

In orthopteran insects, a complex tibial organ has evolved to detect substrate vibrations and/or airborne sound. Species of New Zealand weta (Anostostomatidae) with tympanal ears on the foreleg tibia use this organ to communicate by sound, while in atympanate species (which communicate by substrate drumming) the organ is unstudied. We investigated the complex tibial organ of the atympanate ground weta, *Hemiandrus pallitarsis*, for vibration detection adaptations. This system contains four sensory components (subgenual organ, intermediate organ, *crista acustica* homolog, accessory organ) in all legs, together with up to 90 scolopidial sensilla. Microcomputed tomography shows that the subgenual organ spans the hemolymph channel, with attachments suggesting that hemolymph oscillations displace the organ in a hinged-plate fashion. Subgenual sensilla are likely excited by substrate oscillations transmitted within the leg. Instead of the usual suspension within the middle of the tibial cavity, we show that the intermediate organ and *crista acustica* homolog comprise a cellular mass broadly attached to the anterior tibial wall. They likely detect cuticular vibrations, and not airborne sound. This atympanate complex tibial organ shows elaborate structural changes suggesting detection of vibrational stimuli by parallel input pathways, thus correlating well with the burrowing lifestyle and communication by substrate-transmitted vibration.

## Introduction

Different groups of orthopteran insects, including grasshoppers (Caelifera), crickets, tettigoniids and weta (Ensifera), have evolved diverse and complex signalling systems based on the production and reception of sound or substrate vibration^1–5^. New Zealand weta use elaborate cuticular stridulatory structures to produce acoustic signals, most commonly for defense but also in intraspecific signalling^6–10^. Amongst New Zealand weta, large tympanal hearing organs which detect airborne sound have developed in the foreleg tibia of tree weta (*Hemideina*, 8 spp.)^11^, giant weta (*Deinacrida*, 12 spp.)^11^, and tusked weta (*Anisoura*, 1 spp. and *Motuweta*, 2 spp.)^11^. Vibrational signalling is reported for several species of ground weta in the genus *Hemiandrus* ^12^ (14 spp. with several undescribed species)^13^ and giant weta^14^. Presumably detection of substrate vibratory signals involves tarsal and tibial vibration receptor organs.

All New Zealand species of *Hemiandrus* ground weta lack tympanal hearing organs typical in other Ensifera and are presumed to be insensitive to far-field airborne sound^7, 15^. While sensory hairs on the insect body surface may also respond to substrate vibrations^16, 17^ or airborne sound, their response is restricted to near-field sound^18, 19^ or air-currents^20^. Although abdominal drumming can cause audible sounds on artificial substrates, the main channel for local signalling in *Hemiandrus* appears to be vibrational^12^. Ground weta burrow in the soil but abdominal drumming for mate attraction and mating occurs mainly on leaves of shrubs or ferns which transmit the vibrations^12^. The temporal patterns have been compared for selected species but the frequency characteristics, signal amplitudes or signaling range in the vegetation are so far not documented^12, 15^. In addition, males may use vibrational signals to defend territories around their burrows in the soil^7^. The vibrational signals in *Hemiandrus* mediate pair formation through abdominal drumming on the substrate by both sexes^7, 12, 21^. Drumming is apparently restricted to pre-mating behaviour. Vibrational signals differ between the sexes and are likely species-specific^12^. For *H. pallitarsis*, a species common on the North Island of New Zealand, these signals are homogeneous across different populations in contrast to high levels of DNA divergence. This signal coherence highlights the importance of vibrational communication, and contradicts the existence of multiple (cryptic) species^21^.

In Ensifera, several leg sensory organs are sensitive to sound and substrate vibrations^16, 22, 23^. Specifically, elaborate scolopidial organs occur proximad in the tibiae of all three pairs of legs^23, 24^. In tettigoniids and tree weta, these organs are highly similar and are together termed the complex tibial organ^24–26^. The anatomical similarities allow detailed comparisons and homology analysis across Ensifera^23, 24^. The complex tibial organs of tree weta (*Hemideina* spp.) have been studied in detail, where they consist of the subgenual organ (SGO), intermediate organ (IO), and the *crista acustica* (CA) together with an accessory organ (AO)^20, 25, 27, 28^. The SGO is an important detector for substrate vibrations^16, 17, 26, 29^, while the sensilla of the CA are associated with the cuticular tympana and are the main detectors for airborne sound^25, 28, 30^. The tympana and associated tracheal elaborations occur only in forelegs, while the scolopidial organs comprising the complex tibial organ are present in all leg pairs. The neuroanatomical organisation of the complex tibial organ in the tree weta closely resembles that of the tympanate Tettigoniidae^26, 31^ and Prophalangopsidae (*Cyphoderris*)^32^, however the organ has not been studied in the atympanate ground weta species. This raises questions concerning sensory evolution: is the complex tibial organ conserved in evolution, how is it modified by adaptations to different stimuli, and has the prolonged isolation of New Zealand led to differences in the mechanosensors compared to other Ensifera?

The New Zealand weta fauna allows for a comparative investigation of these sensory structures between closely related species of the tympanate tree weta *Hemideina* and the atympanate ground weta *Hemiandrus*. Here, we study the complex tibial organ in *Hemiandrus pallitarsis*, an atympanate species. In addition to the above general questions, we focus on similarities and differences in neuroanatomy relating to vibrational detection in the absence of elaborate tympanal hearing organs, and specifically on possible changes in the sensilla of the CA. For the component sensory organs, we document the sensillum numbers and the serial organisation for possible specialisations between leg pairs. An important aspect of the complex tibial organ are morphological adaptations, in the component scolopidial organs, which mediate the mechanical coupling between different stimuli and the respective neuronal sensilla. Therefore, we investigate the attachments of the different sensory organs and their neurons within the tibia. We then compare these findings to the complex tibial organ anatomy of *Hemideina* legs. The aim is to seek sensory adaptations which preferentially allow insects to detect either airborne sound or substrate vibration, in closely related insects with tympanate and atympanate sensory organs. We show, for the first time, distinct attachments of the atympanate mechanosensory organs which provide parallel input pathways for vibration stimuli.

## Materials and Methods

### Study animals

**Figure 1:**
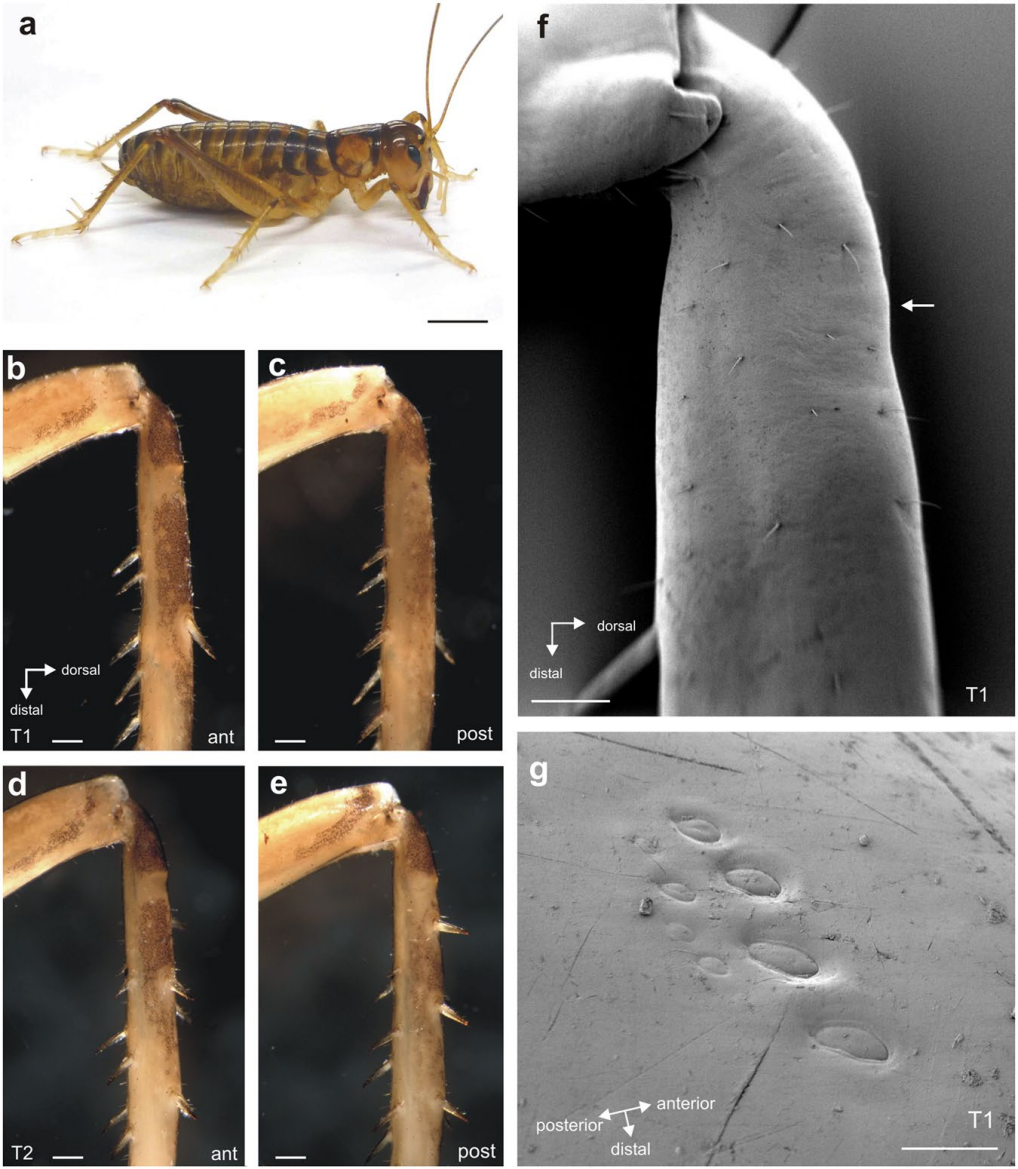
*Hemiandrus pallitaris* and external leg morphology. (**a**) Female individual of *H. pallitarsis*. Photograph by J. Strauß. Forelegs lack tympanal membranes on the anterior (**b**) or posterior face (**c**), and are similar to the midlegs (**d**,**e**). Note the proximal tibial areas with less pigmentation on both sides of the leg. Cuticle is generally solid with no vestiges of tympanal membranes, seen in the scanning electron micrograph (**f**). White arrow indicates the location of campaniform sensilla shown in (**g**). (**g**) Two rows of campaniform sensilla are located on the dorsal foreleg. Abbreviations: ant, anterior; post, posterior. Scales: (**a**) =7.5 mm. (**b**–**e**) =500 µm; (**f**) =250 µm; (**g**) =20 µm.

Female and male *H. pallitarsis* (Walker, 1871) (Fig. 1a) were collected from foliage in a riparian habitat on Massey University campus in Palmerston North, New Zealand (for neuroanatomical studies) or on Little Barrier Island, New Zealand (for tracheal reconstruction) (New Zealand Department of Conservation permit number WK-29320-FAU). The weta were kept in separate plastic containers containing soil. Animals were fed grain on a daily basis and provided with water from small glass tubes *ad libitum* (neuroanatomy study) or provided with a diet of meal worms and fish food flakes *ad libitum*. They were kept in the laboratory at room temperature (trachea study).

Male and female *Hemideina thoracica* (Walker, 1846) were collected from artificial galleries (diurnal shelters) located in native bush areas around Auckland, New Zealand. Weta were kept in an enclosure with a diet of New Zealand native plant material and fish food flakes. All experiments followed the guidelines of the University of Canterbury Animal Ethics Committee (AEC) Code of Ethical Conduct as laid down by the Animal Welfare Act 1999.

### Scanning Electron Microscopy

External structures of specimens stored in 70% ethanol were documented by scanning electron microscopy. We analysed foreleg tibiae and the first spiracle in the thorax. Legs and pieces of cuticle around the first thoracic spiracle were cut with scissors and were dehydrated in a graded ethanol series. The preparations were critical point-dried (BAL-TEC, Balzers, Liechtenstein) and sputter-coated with gold (SCD 004 SputterCoater, Balzers). They were viewed and documented with a Phillips XL 20 SEM.

### Thoracic tracheation

The tracheal system in the prothorax was investigated by dissection of freshly killed individuals. The animal was pinned out in a glass dish filled with Sylgard (Sylgard 184, Suter Kunststoffe, Fraubrunnen, Switzerland). The prothorax was then carefully cut open on the ventral side under locust saline and the trachea was exposed and drawn using a Wild dissection binocular microscope with a camera lucida.

### Three-dimensional reconstruction and image processing of trachea, tracheal auditory vesicles and internal tissues

To construct a 3D model of the tibial tracheae from both *H. pallitarsis* and *H. thoracica* the foreleg was removed at the coxa and immediately fixed in 70% ethanol. Each leg was scanned using an in-house micro-CT system based on a Feinfocus microfocus x-ray tube (spot size ~5 um) and a Photonic Science X-ray camera (CSIRO, Melbourne Australia). The scanned stacks of images were imported in AVISO3D analysis software (v.9.0.1., FEI, Hillsboro, Oregon). The tracheae were segmented and converted into labels using the AVISO Lasso freehand auto trace tool. To smooth out the label and reduce the step effect of segmentation, a Gaussian filter was applied. A surface view was generated of both the tracheae label and external leg label. The external leg label was made transparent to show the orientation of the tracheae within the tibia.

### Axonal tracing studies

The neuroanatomy of the complex tibial organ was documented by retrograde axonal tracing. Two leg nerves supply sensory organs and muscles in the tibia, termed N5B1 (main sensory nerve) and N5B2 (main motor leg nerve)^28^. Isolated legs from all three thoracic segments were fixed with insect pins in glass dishes filled with Sylgard. The femur was cut open with a piece of a blade and the nerves exposed. Either N5B1 or both N5B1 and N5B2 were prepared for tracing by cutting with iridectomy scissors proximally before the femur-tibia joint. The free nerve ends were transferred into glass capillaries filled with a solution of 5% CoCl_2_ dissolved in locust saline. Preparations were incubated at 4 °C overnight in a moist chamber. After the incubation period, the tarsi and most of the femur were cut off the legs which were then transferred into locust saline. Intracellular cobalt was precipitated by incubation in 1% ammonium sulphide in locust saline for 10–15 min. The legs were fixed in Delbucco’s fixative for 60 min, dehydrated in a graded ethanol series (at 30, 50, 70, 90, 96, 100%, 60 min per step) and finally cleared in methyl salicylate (Fluka, Buchs, Switzerland) over night.

### Light microscopy

Intact legs from specimens stored in ethanol were photographed using a Leica DCF-320 camera (2088 × 1055 pixels) on a Leica dissection microscope.

Backfill preparations were analysed with an Olympus BH-2 microscope, and photographed with a Leica DCF-320 camera (2088 × 1055 pixels). Most preparations were photographed in series, and stacked images were generated using the freeware program CombineZP (http://www.hadleyweb.pwp.blueyonder.co.uk).

Drawing reconstructions of sensory organs and their innervation were made by analysis with a Leitz Dialux microscope with a Leitz drawing tube or from an Olympus BH-2 microscope with a camera lucida.

### Confocal laser-scanning microscopy

Specimens previously preserved and cleared in methyl salicylate (as above) were returned to 100% ethanol for 2 × 30 min washes, and then transferred to 0.05% eosin-Y in 100% ethanol for 45 min. They were de-stained in two washes of 100% ethanol (50 and 30 min) and transferred to 100% methyl salicylate. Z-stacks of 2 μm thick optical sections were obtained with a Leica SP5 inverted confocal scanning microscope, using a 10x objective with the laser excitation at λ = 514 nm and emission at λ = 540–650 nm. Stacked images were successfully obtained from fore- and midlegs and were processed with Google Picasa to produce videos to allow scanning and analysis of internal tibial structure from posterior to anterior sides. The eosin provided general staining of background soft tissue as well as strong staining of neuronal somata and dendrites. To reconstruct 3D models of the confocal scans, the Z-stacks were imported and rendered in AVISO (described above) and Drishti (Drishti v 2.6.1,The Australian National University, Canberra, Australia) using a 2D transfer function.

### Neuroanatomy of the sensory neuropil in ganglion sections

Ganglia with anterograde tracing were embedded in Agar100 epoxy resin (Plano, Wetzlar, Germany) following the producers’ instructions. Ganglia were sectioned with a Leica RM 2165 microtome at 7 µm thickness. The resulting sections were counterstained with methylene blue (Serva, Heidelberg, Germany; 1% dissolved in distilled water). They were then embedded in Entellan (Roth Chemicals, Karlsruhe, Germany) for microscopy.

### Documentation

Panels of photomicrographs were assembled and labelled using CorelDraw 11 (Corel; Ottawa, Canada). Individual photographs were slightly adjusted for brightness and contrast using Corel PhotoPaint (Corel, Ottawa, Canada).

### Statistical analysis

Statistical analysis was carried out using the software Prism 4 (GraphPad, San Diego, CA). Numbers of sensilla in the sensory organs were tested for normal distribution using the Kolmogorov-Smirnov normality test, and consecutively analysed for data not normally distributed with the Kruskal-Wallis test with Dunn’s Multiple Comparison *post hoc* test, or for data with normal distribution with the one-way ANOVA with Tukey’s Multiple Comparison test.

## Results

### External leg anatomy

The tibiae of *H. pallitarsis* had solid cuticle lacking obvious tympanal membranes or tympanal vestiges in the proximal tibia (Fig. 1b–f). There were no notable differences in the cuticle between foreleg and midleg (Fig. 1b–e). Unpigmented areas in the narrower proximal part of the tibia occurred on both anterior and posterior sides, and this pigment distribution was similar in forelegs (Fig. 1b,c), midlegs (Fig. 1d,e) and hindlegs (Figs 1a and 4a). A group of approximately 8 campaniform sensilla was located on the dorsal side of the tibia. The cuticular caps of those campaniform sensilla in the anterior row were larger in diameter than those in the posterior row (Fig. 1g).

### Spiracle and tracheal structures

**Figure 2:**
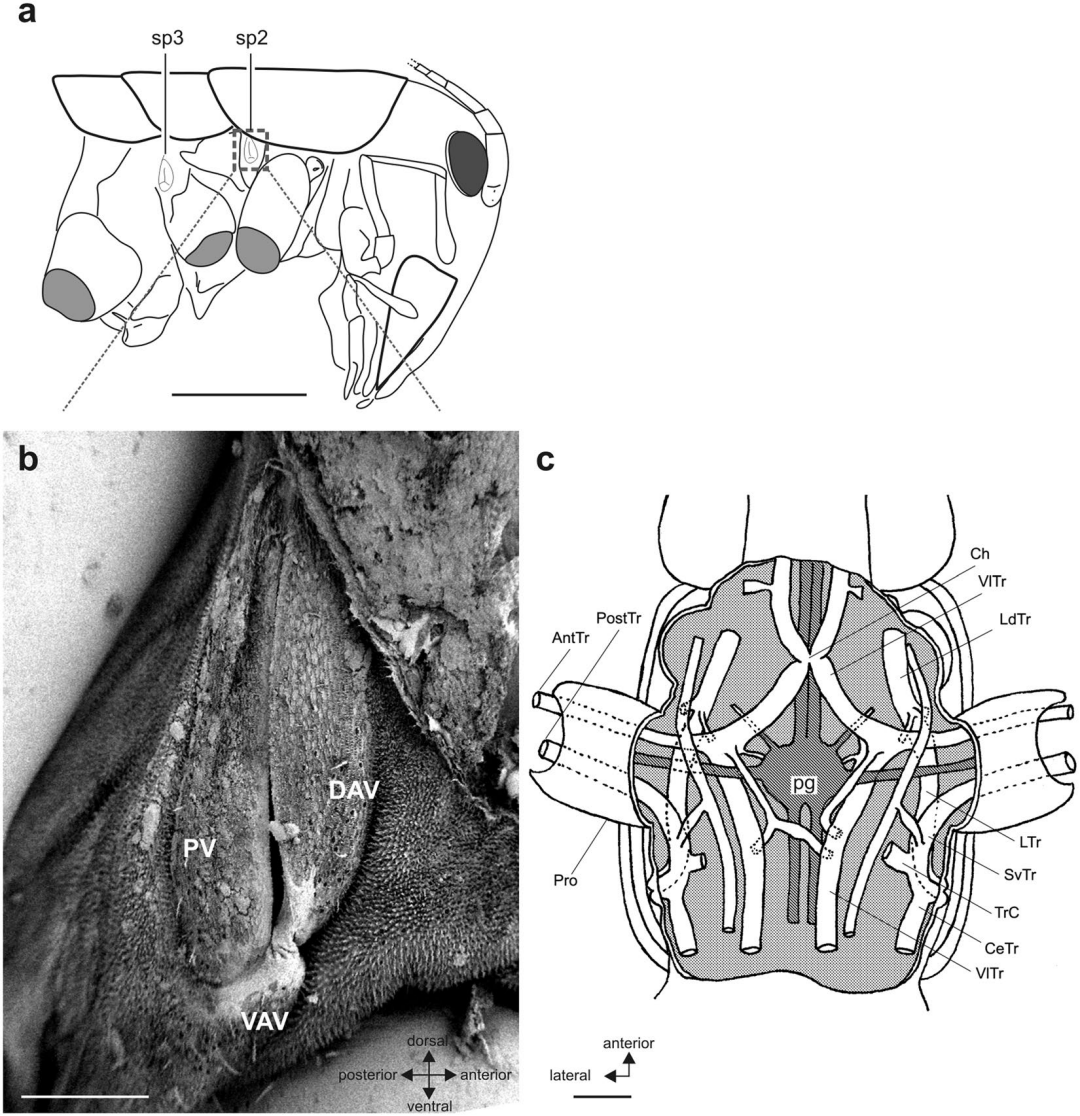
Spiracle and tracheal system in the prothoracic segment. (**a**) Overview indicating the spiracle in the prothoracic (sp2) and mesothoracic (sp3) segment. Box area in (**a**) is shown in (**b**). (**b**) Scanning electron micrograph of the elongated spiracle in the prothoracic segment which is closed by three valves. **(c)** Schematic of the tracheal system in the prothorax (ventral view). Two tracheae (AnTr, PostTr) enter the prothoracic leg (Pro). Nerves originating from the prothoracic ganglion (pg) are simplified. Abbreviations: AntTr, anterior trachea, CeTr, cephalic trachea; Ch, tracheal chiasma; DAV, dorsal anterior valve; LdTr, laterodorsal trachea; LTr, lateral trachea; pg, prothoracic ganglion; PostTr, posterior trachea; Pro, prothoracic leg; PV, posterior valve; SvTr, supraventral trachea; TrC, transverse commissure; VAV, ventral anterior valve; VlTr, ventral longitudinal trachea. Scales: (**a**) =5 mm; (**b**) =250 µm; (**c**) =500 µm.

In the thorax, spiracles located in the prothoracic and mesothoracic segment (Fig. 2a), were termed spiracle 2 (sp2: prothorax) and spiracle 3 (sp3: mesothorax)^33^, as they originate in the respective posterior segments during ontogeny. The spiracle 2 in the prothorax was covered by three valves (Fig. 2b): the dorsal anterior, ventral anterior, and posterior valve, which together closed the spiracle. This spiracle was elongated in the dorso-ventral axis. The ventral anterior valve was rather small compared to the two other valves (Fig. 2b). The spiracle 2 showed no potential acoustic specialization, and its aperture was similar to that of the spiracle in the mesothorax.

**Figure 3:**
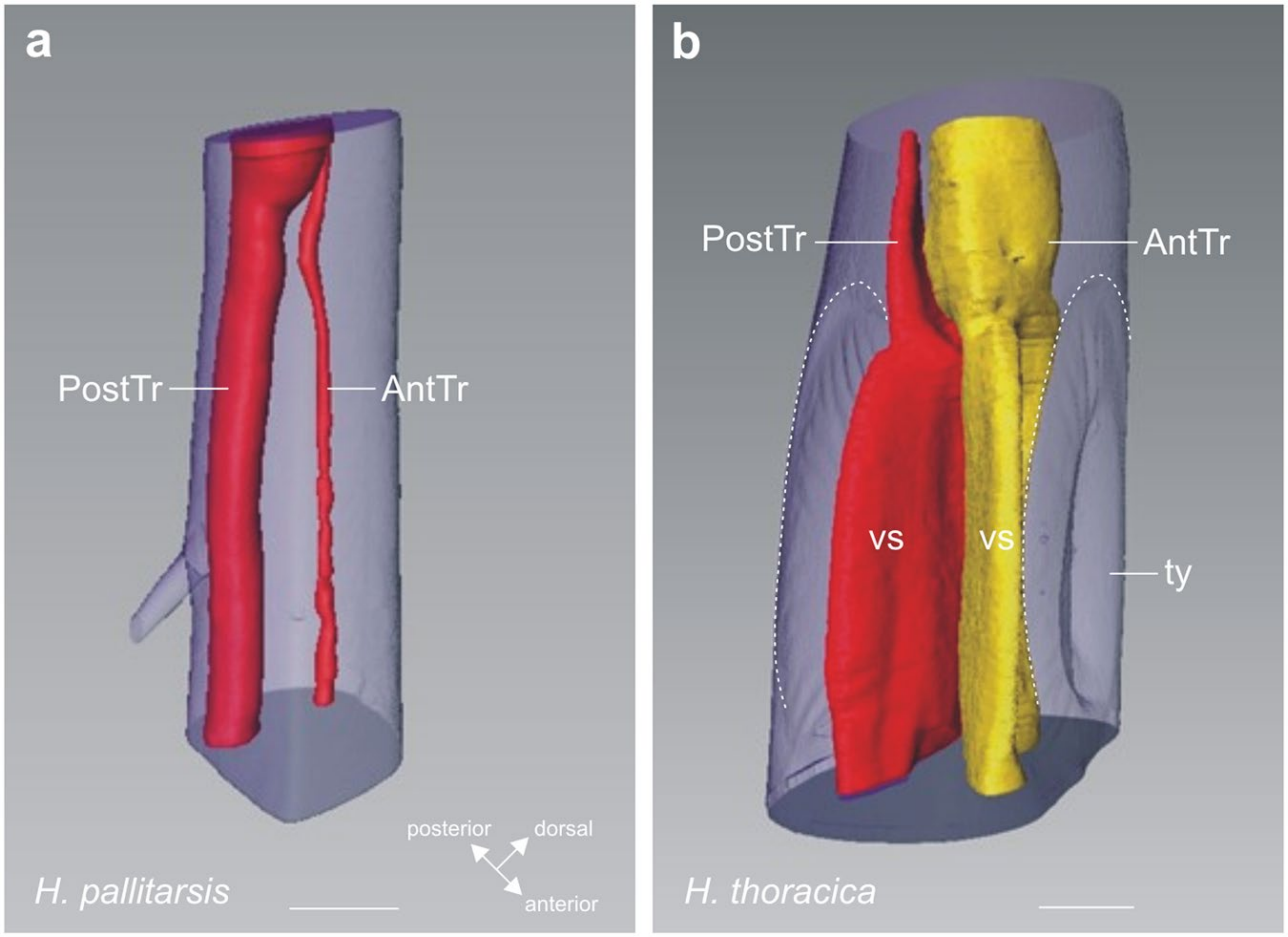
Tracheal system in the foreleg tibia reconstructed from μCT scans. Tibial trachea in (**a**) ground weta *H. pallitarsis* and (**b**) the tree weta *H. thoracica*. An anterior (AntTr) and posterior tibia (PostTr) run in the tibia, which are enlarged in the tree weta between the tympanal membranes (ty; hatched lines) as acoustic vesicles (vs). Abbreviations: at, anterior trachea; pt, posterior trachea; ty, tympanum; vs, vesicles. Scale: (**a**) =200 µm, (**b**) =500 µm.

In the prothorax, three tracheae originated from the spiracle: the cephalic trachea, the lateral trachea and the supraventral trachea (Fig. 2c). The supraventral trachea entered along the posterior side of the foreleg. It is termed the posterior trachea in the femur and tibia. The lateral trachea connected with a branch of the ventral longitudinal trachea (VlTR) running laterally and entering the foreleg. This VlTr branch formed the anterior trachea running through the tibia (Fig. 2c). In the tibia, these two tracheae run close to each other (Fig. 3a). The proximal posterior trachea was enlarged compared to the anterior trachea. In both tracheal branches no significant change in size was notable along the proximo-distal axis. In comparison, the two tibial tracheae in *Hemideina thoracica* formed greatly enlarged and flattened tympanal vesicles adjacent to each tympanum (Fig. 3b), and distally the posterior trachea formed a large chamber which enhances hearing sensitivity^25, 34^. The tracheal vesicles indicated the importance of the tracheal system for hearing in the tree weta. However, such adaptations were absent in ground weta. In sum, the ground weta lacked the obvious adaptations for an auditory system: tympana, open spiracle, and tibial tracheae enlarged into acoustic vesicles.

### Neuroanatomy of the complex tibial organ

**Figure 4:**
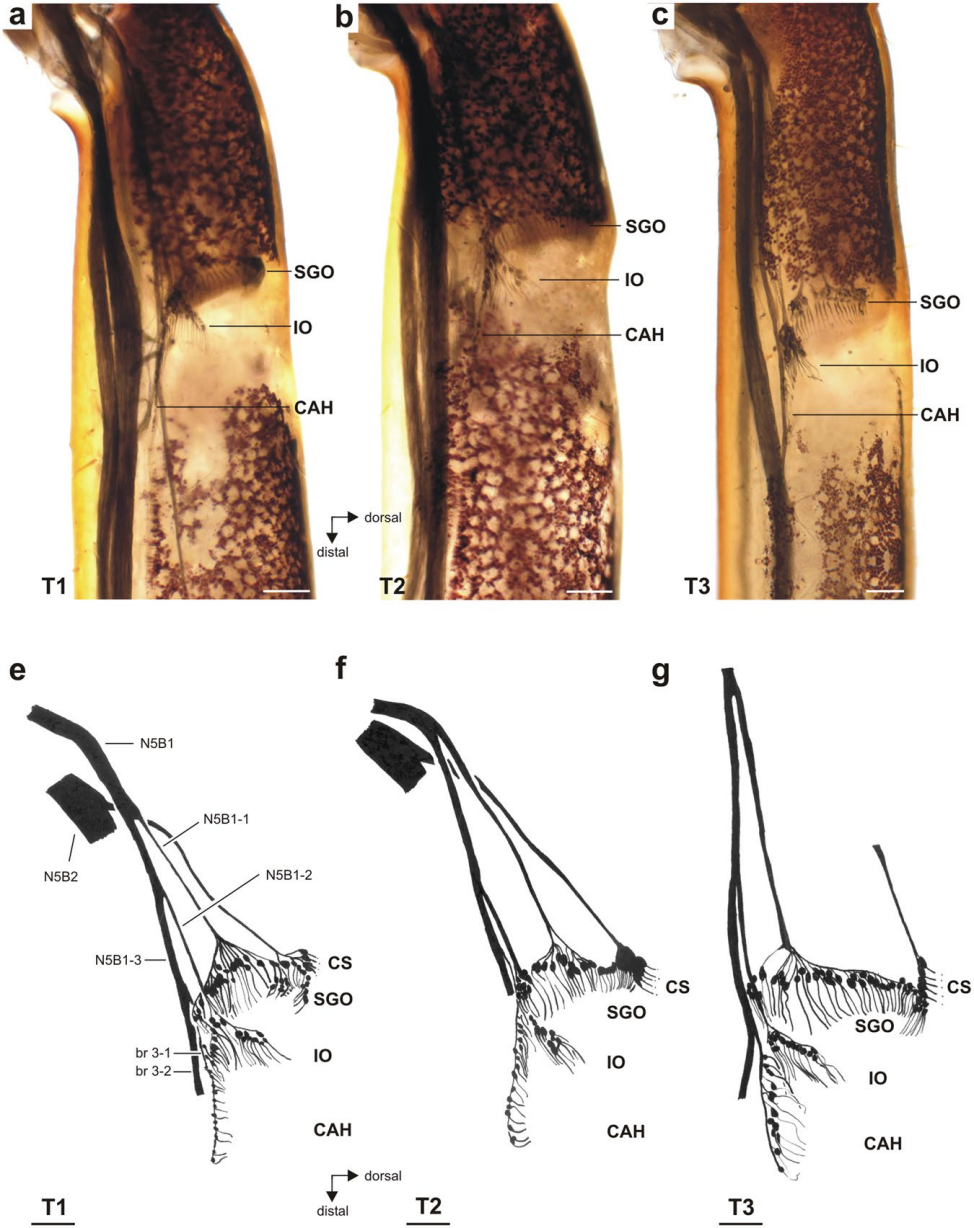
Whole mount preparations and schematic of the complex tibial organ in all three leg pairs. (**a**–**c**) In all legs, the subgenual organ (SGO), intermediate organ (IO) and *crista acustica* homolog (CAH) are present. Viewed from anterior. Note the lack of pigmentation in the cuticle at the level of the sensory organs. (**d**–**f**) Drawings of the complex tibial organ and its innervation in serial organisation. Anterior perspective. The structure and innervation of sensory organs were highly similar in all three leg pairs. Innervating nerves and nerve branches are labelled for the foreleg. The accessory organ, which is also innervated by N5B2, has been omitted for clarity. For the hindleg T3, the nerve N5B2 is omitted. Abbreviations: CAH*, crista acustica* homolog; CS, campaniform sensilla; IO, intermediate organ; SGO, subgenual organ; T1, foreleg; T2, midleg, T3, hindleg. Scale = 100 µm.

**Figure 5:**
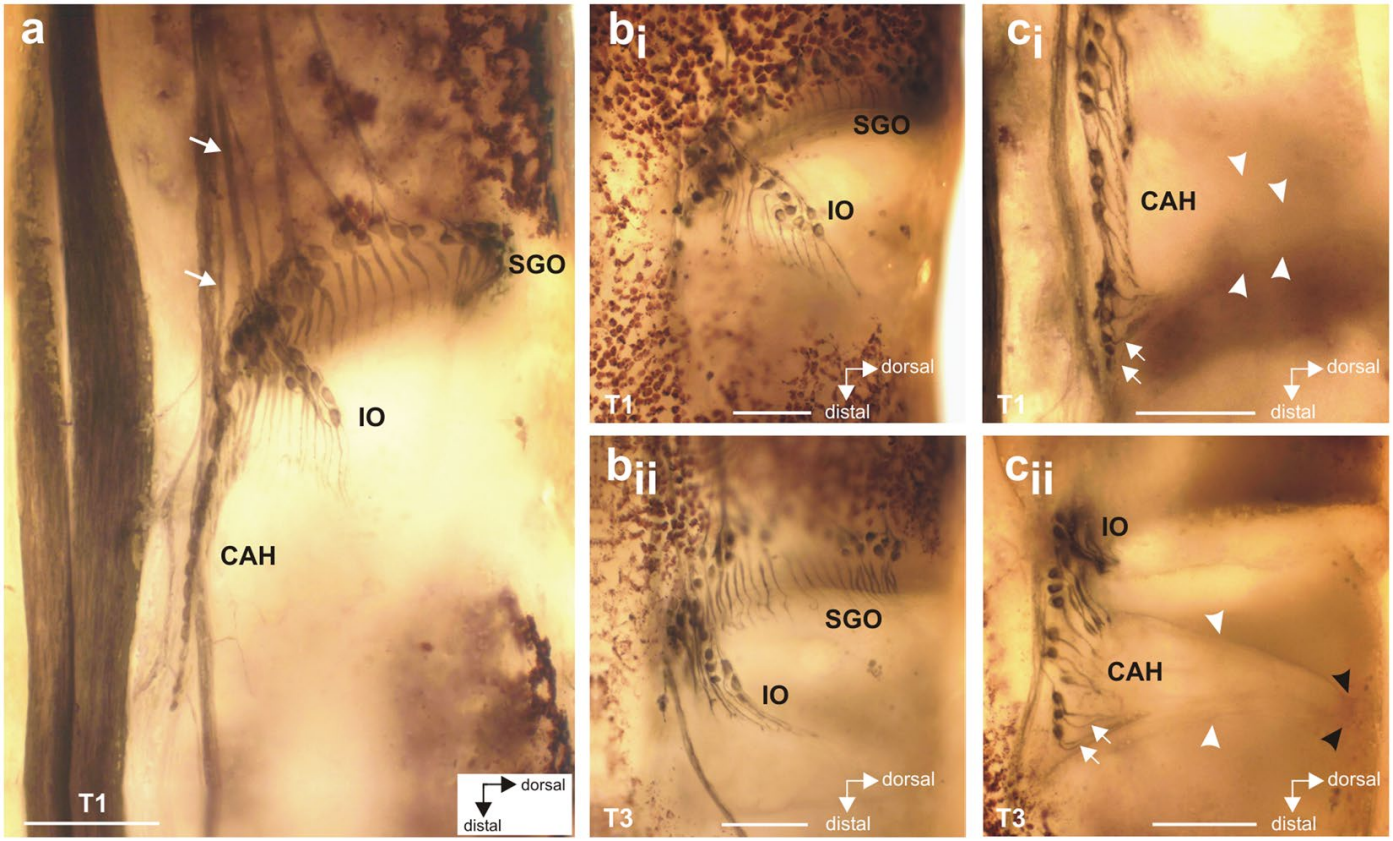
Details of the intermediate organ and *crista acustica* homolog in the foreleg. (**a**) Lateral view of the complex tibial organ. The distinct nerves innervating the intermediate organ and the *crista acustica* homolog are indicated by arrows. (**b**) Lateral view of the intermediate organ in the foreleg (bi) and hindleg (bii). (**c**) Lateral view of the *crista acustica* homolog in foreleg (ci) and hindleg (cii) showing the linear arrangement of somata. Dendrites of the distal CAH sensilla bend steeply dorsally (white arrows). The IO and CAH sensilla are attached to a triangular mass of connective tissue and attachment cells (white arrowheads) which tapers dorsally (black arrowheads) and is attached to the anterior cuticle along its entire length. Abbreviation: CAH, *crista acustica* homolog; IO, intermediate organ; SGO, subgenual organ; T1, foreleg; T3, hindleg. Scale: (**a**) =100 µm; (**b**), (**c**) =50 µm.

**Figure 6:**
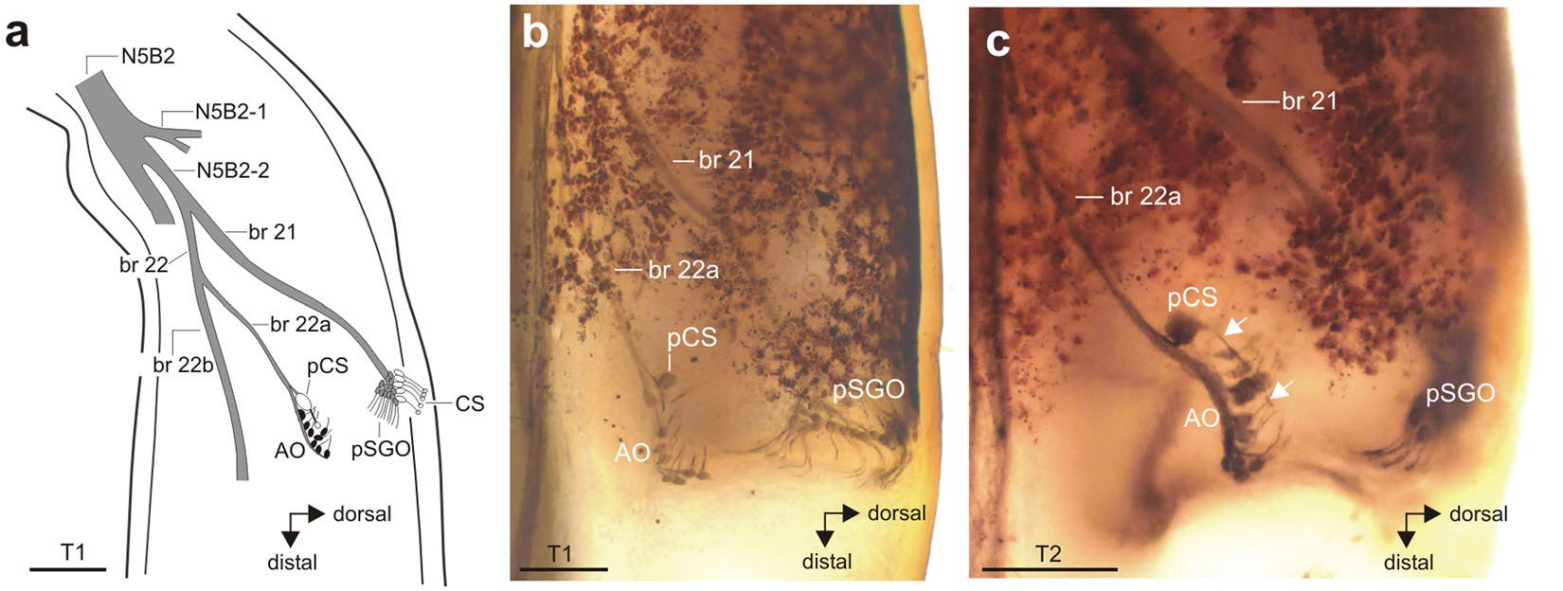
The structure and innervation of the accessory organ (AO). (**a**) Schematic of the AO and its innervation by the nerve N5B2. Different nerve branches are numbered consecutively from the proximal direction. Sensilla in the AO are shown in black, sensilla in the SGO in grey, and campaniform sensilla are shown in white. The AO shows similar organisation in (**b**) foreleg T1 and (**c**) midleg T2. The scolopidial sensilla of the accessory organ are always associated with a single posterior campaniform sensillum (pCS), the long dendrite of the pCS is indicated by two white arrows in (**c**). Note that AO dendrites point in proximal or proximo-dorsal direction. Abbreviations: AO, accessory organ; CS, dorsal campaniform sensilla; pCS, posterior campaniform sensillum; pSGO, posterior subgenual organ. Scales: (**a**,**b**) =100 µm; (**c**) =200 µm.

Axonal tracing stained the complex tibial organ and associated innervation in the proximal tibia (Fig. 4a–c). These were located at the level of the faintly pigmented tibial cuticle (Figs 1 and 4a–c) which made the organs visible for analysis even in whole-mount preparations of all three leg pairs. The complex tibial organ was innervated mainly by the predominantly sensory nerve N5B1, and this nerve innervates three scolopidial organs: the subgenual organ (SGO), intermediate organ (IO), and the *crista acustica* homolog (CAH) (Fig. 4). These organs in *H. pallitarsis* could be distinguished by the different spatial arrangement of sensilla, and also by neuronal innervation by separate nerve branches from N5B1 (Figs 4 and 5a). The nerve N5B2 innervates the posterior SGO, campaniform sensilla, and the accessory organ close to the posterior SGO (Figs 4 and 6). The SGO is thus innervated by two different nerves. The overall organization is highly similar to the complex tibial organ in the tree weta *Hemideina*, which also contains four scolopidial organs^25, 28^.

Among the sensory organs, the SGO contained the highest number of scolopidial sensilla (~50), which were arranged in a fan-like array (Table 1). Higher numbers of sensilla were counted in the SGO and IO of the fore- and midleg than in the hindleg (Table 1; Supplementary Data 1). These differences for both organs were statistically significant, and for both organs they depended on the lower sensillum numbers in the hindleg (SGO: Kolmogorov-Smirnov test: p > 0.1 for all leg pairs; one-way ANOVA: p = 0.0034; F = 7.548; R^2^ = 0.4182; Tukey’s Multiple Comparison test: T1 vs. T3: p < 0.05; T2 vs T3: p < 0.01; IO: Kolmogorov-Smirnov test: p > 0.1 for all leg pairs; one-way ANOVA: p = 0.0011; F = 9.253; R^2^ = 0.4459; Tukey’s Multiple Comparison test: T1 vs. T3 and T2 vs. T3: p < 0.01).

**Table 1:** Number of scolopidial sensilla in the organs of the complex tibial organ in *H. pallitarsis*. Abbreviations: T1, foreleg; T2, midleg; T3, hindleg.

	Subgenual organ	Intermediate organ	*Crista acustica* homolog	Accessory organ
T1	50 ± 4 (n = 9)	18 ± 2 (n = 9)	12 ± 2 (n = 9)	10 ± 2 (n = 9)
T2	52 ± 3 (n = 8)	19 ± 2 (n = 8)	13 ± 2 (n = 8)	10 ± 2 (n = 9)
T3	46 ± 7 (n = 6)	15 ± 3 (n = 9)	11 ± 1 (n = 9)	9 ± 2 (n = 4)

Most SGO sensilla were innervated by N5B1-1, and their dendrites were mainly oriented in a distal direction (Fig. 4). Only the posterior SGO and the accessory organ were innervated by N5B2 (Figs 4d–f and 6). SGO sensilla occured closely packed and the precise transition between groups with innervation from these two nerves was difficult to identify (Fig. 4). The dendrites of these posterior SGO sensilla bent in a ventro-distal direction (Figs 4d–f and 6). The posterior SGO sensilla were closely located to the cluster of somata of the dorsal campaniform sensilla (Figs 1g and 4d–f).

The groups of sensilla in the IO and CAH were innervated exclusively from N5B1 by distinct nerve branches, termed N5B1-2 for the IO, and N5B1-3-1 for the CAH (Figs 4 and 5). The IO was located distally to the SGO. The sensilla of the IO extended prominently in the dorsal direction of the tibia (Figs 4 and 5b). The CAH was located in the distal-most position in the complex tibial organ. The CAH sensilla were arranged in a line which commenced close to the base of the IO (Figs 4 and 5). There were on average 11–13 sensilla in the CAH among leg pairs (Table 1), and the numbers of sensilla were similar between the thoracic legs. The sensillum numbers in the CAH were analysed for differences between leg pairs after they were tested for normal distribution (Kolmogorov-Smirnov normality test; T1: p > 0.1; T2: p > 0.1; T3: p = 0.0021). Since the hind leg data were not normally distributed, we further tested for differences between leg pairs with the Kruskal-Wallis test. The sensillum numbers between the different leg pairs were not statistically significant (Kruskal-Wallis test; p = 0.3056; Kruskal-Wallis statistic = 2.371; Dunn’s Multiple Comparison Test: p > 0.05 for comparisons T1–T3). This indicates that no obvious sensory specialisation in sensillum numbers exists in the CAH between the leg pairs.

Another small scolopidial organ was located at the posterior side of the tibia, next to the posterior SGO closely under the leg cuticle (Fig. 6). This organ is termed the accessory organ (AO) in tree weta^25, 34^ and other Ensifera^35^. The accessory organ was present in all three leg pairs in *H. pallitarsis*. The sensilla were clearly spatially separated from the SGO sensilla, and were also innervated by a distinct nerve branch from N5B2 (Fig. 6a). The AO contained on average 9–10 scolopidia (Table 1) always associated with a single posterior campaniform sensillum with a large soma and a rather long dendrite (Fig. 6). The AO showed no anatomical differences between leg pairs, and sensillum numbers were not significantly different between leg pairs (Kolmogorov-Smirnov test: p > 0.1; one-way ANOVA: p = 0.3396; F = 1.144; R^2^ = 0.1074).

In sum, we found high similarities between the neuronal elements of the complex tibial organ of the ground weta *Hemiandrus* and the tree weta, *Hemideina*. Despite the atympanate external anatomy in the ground weta, the complex tibial organ is as elaborate as that of the fully tympanate tree weta with SGO, IO, CAH and AO. These different organs have specific positions, sizes, and orientation of sensilla.

### Attachment of the sensory organs

**Figure 7:**
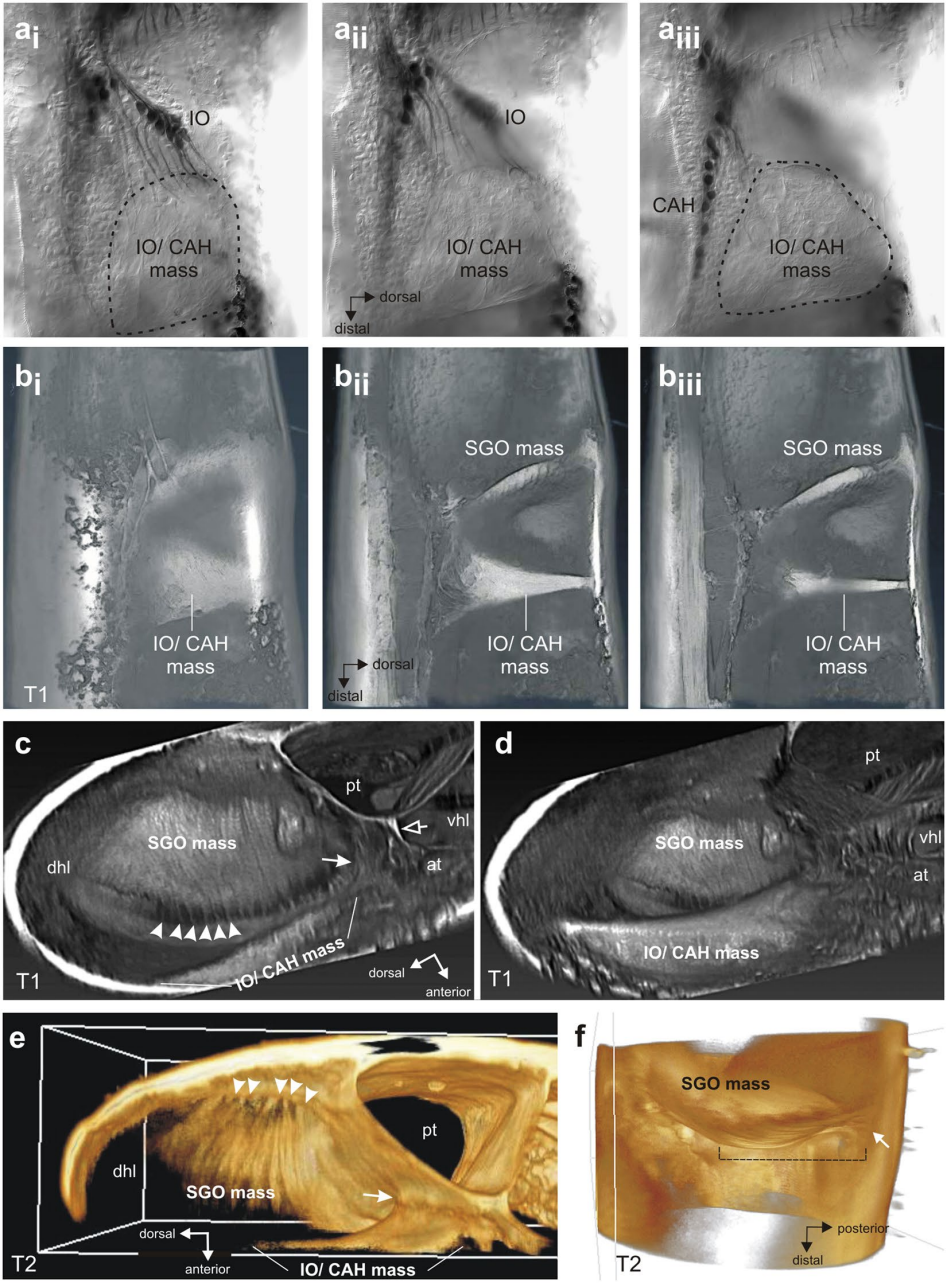
Attachment elements of the sensory organs in the tibia. (ai–iii) Phase contrast micrographs of CTO viewed from anterior showing insertion of IO dendrites in the IO/CAH mass. Sections move from anterior tibial wall (ai) to medial region of tibia (aiii). Note different planes of dendrite innervation into the IO/CAH mass for the IO and CAH. (**b**) Reconstruction of tibia from confocal stack of lateral (anterior) view of eosine-stained preparation showing the morphology of the SGO and IO/CAH-masses. Sections move from anterior to medial, showing the thinning of the IO/CAH mass. (**c**) µCT reconstruction of the foreleg tibia in transverse section, looking proximally and showing the SGO mass spanning the tibia with fine suspensory extensions along the anterior margin (white arrowheads). The IO/CAH mass is linked to the tracheae by connective tissue (white arrow). A thin septum connects the anterior and posterior tracheae (empty arrow) and separates the dorsal and ventral hemolymph channels. (**d**) µCT reconstruction of the foreleg tibia in transverse section at the level just distal to that in (**c**). The thick IO/CAH mass is attached along its full length to the anterior tibial wall, but shows only indirect association with tibial tracheation. (**e**) µCT of the midleg tibia. The elongated lamellar structures across the surface of the SGO mass attach to the posterior tibial wall via fine extensions (white arrowheads), as revealed by digital erosion of overlying tissue. Part of the IO/CAH mass is connected to the posterior trachea (white arrow). (**f**) Transverse view of the SGO with overlying tissue eroded away to show the lamellar layer (hatched line) which gives rise to thin attachment strands on the anterior side and posterior side of the tibial wall (shown here; arrow). Abbreviations: at, anterior trachea; CAH, *crista acustica* mass; dhl, dorsal hemolymph channel; IO, intermediate organ; pt, posterior trachea; SGO, subgenual organ; SGO mass, subgenual organ mass; vhl, ventral hemolymph channel.

Sensilla of the CTO were placed in two distinct masses or attachment sites: the disk-like SGO mass (Fig. 7) and the thick triangular IO/CAH mass (Figs 5 and 7). µCT reconstruction revealed that the SGO mass spans the dorsal hemolymph channel in the proximal end of the tibia (Fig. 7c,d). Its thick posterior margin was firmly attached around the dorsal and posterior cuticle while the thin anterior margin gave rise to many short, fine extensions that attached to the anterior and posterior cuticle (Fig. 7c,e,f). These created openings through which hemolymph can pass, and hence the SGO did not fully occlude the hemolymph channel. The SGO mass was not homogeneous as the distal surface was covered by parallel, strap-like structures each of which arose from the fine attachment strands and extended posteriorly across the surface of the SGO mass (white arrowheads, Fig. 7c–e). By using an FFT erosion filter to digitally remove overlying tissue in the µCT reconstruction, it was discovered that the straps form a distinct lamellum (Fig. 7f) which extends, and attaches to, the posterior tibial wall as well (Fig. 7e,f). These straps terminate in the same fine filaments seen on the anterior aspect of the SGO, but on the posterior tibial side they are embedded in SGO tissue and therefore do not allow hemolymph flow along this side.

The IO/CAH mass arose distal to the SGO mass, as a broadly spread structure fully anchored onto the anterior tibial wall from the midline to the dorsal extent of the hemolymph cavity (Fig. 7bi). Its thickness decreased medially (progression: Fig. 7bi–biii), forming an elongated triangular surface with the apex at the dorsal cuticle and the base anchored in the region of the tracheae in the medial midline. In transverse tibial section it formed a roughly crescentic outline, as suggested in the AVIRA reconstruction of confocal images (Fig. 7d). The dendrites from the IO extended distally into the IO/CAH mass in a region of large elliptical cells adjacent to the cuticle (Fig. 7ai). It is not clear whether these were cap (attachment) cells. The CAH dendrites lay in a more medial plane than those of the IO (compare Fig. 7ai and aiii), and they inserted sequentially along the base of the IO/CAH mass. They pointed dorso-distally, except for the most distal ones which are reflected proximally (Fig. 5ci,ii).

In tympanate ensiferans, the IO and CA are intimately associated with the highly modified anterior tibial trachea to permit acoustic sensitivity. In *H. pallitarsis*, the IO/CAH mass was not closely attached to the anterior trachea, as it lay between the anterior and posterior trachea with intervening connective tissue. There was a minor connection of the IO/CAH mass to the posterior trachea, but no structurally prominent coupling (Fig. 7b,c,d). The shared attachment for the IO and CAH made the organs likely a functional unit with indirect attachments to the tibial tracheae and direct attachment to the anterior tibial cuticle.

In terms of functional morphology, the SGO, IO/CAH and AO thus differ in their size, their orientation in the leg, and importantly their attachments, indicating different functional specialisations. In particular, the complex attachments of the SGO, and the unexpected discovery of a positional shift of IO/CAH from the tibial hemocoel to the cuticular wall, strongly point to different modes of stimulation. The exposure of the IO/CAH mass to hemolymph movements is much more restricted than for the SGO mass, making induced movements from cuticular vibrations the most likely stimulus source for the IO/CAH.

### Central projection of sensory afferents

**Figure 8:**
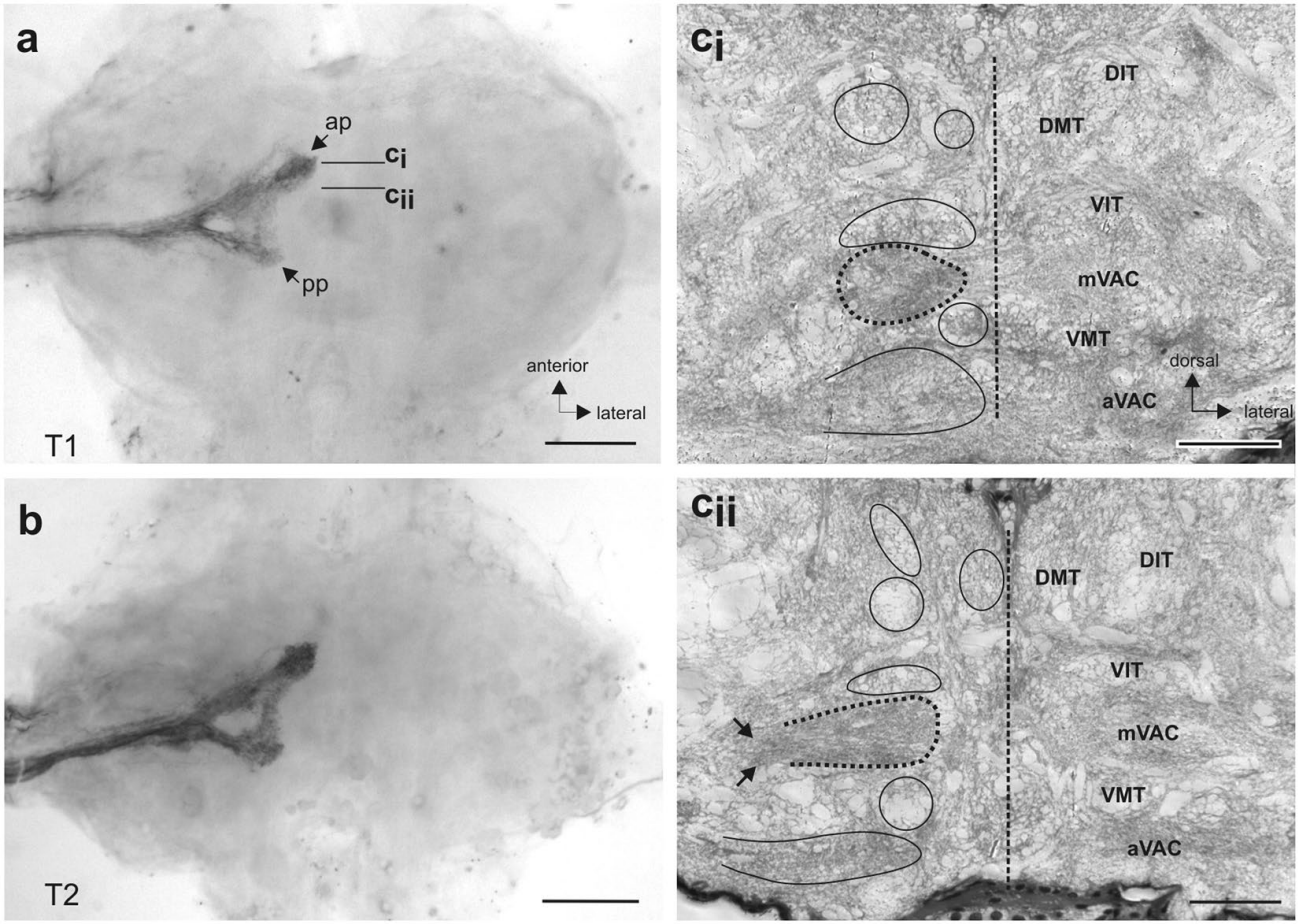
Central projection of the complex tibial organ afferents. Staining of sensory afferents in N5B1 in the (**a**) prothoracic ganglion and (**b**) mesothoracic ganglion. Afferents terminate on the ipsilateral side in anterior and posterior projections (ap/pp). (ci–ii) Histological sections of the prothoracic ganglion locate the afferents in the main mechanosensory neuropil, the mVAC (indicated by dotted circle). Sections show the main neuropils and tracts (encircled) in the ganglion. Levels of sections are indicated in (**a**). Arrows in (cii) indicate the bundle of fibres entering the mVAC. Abbreviations: ap, anterior projection; aVAC, anterior ventral association centre; DIT, dorsal intermediate tract; DMT, dorsal median tract; mVAC, median ventral association centre;pp, posterior projection; T1, prothoracic ganglion; T2, mesothoracic ganglion;VIT, ventral intermediate tract; VMT, ventral median tract. Scales: (**a**,**b**) =200 µm; (**c**,**d**) =100 µm.

The projections of sensory afferents were documented by axonal tracing of N5B1 into the respective ganglion for foreleg and midleg. The projections entered the ganglion as a bundle of fibres which extended close the ganglion midline (Fig. 8). The afferents remained strictly ipsilateral in the prothoracic and mesothoracic ganglion (Fig. 8a,b). Within the ganglion, the afferents separated into an anterior and posterior projection of similar sizes, but with the anterior projection closer to the ganglion midline (Fig. 8a). There were no obvious differences in the central projection between prothoracic (Fig. 8a) and mesothoracic ganglion (Fig. 8b). Histological sections of the prothoracic ganglion revealed the neuropils in which stained afferents terminate. Sections from the anterior projection of N5B1 terminated in the medial ventral association centre (mVAC) (Fig. 8c). The mVAC is not located directly at the midline and is not fused between the ganglion hemispheres.

## Discussion

### Neuroanatomy of the complex tibial organ of the atympanate *Hemiandrus pallitarsis*

The aim of this study was to compare the sensory structures in the complex tibial organ of ground weta to those of the closely related tympanate tree weta. The overall organisation of the complex tibial organ of *H. pallitarsis* was identical to that in related tympanate *Hemideina* tree weta species. In closer detail however, differences occured in sensillum numbers and arrangement between genera.

The numbers of sensilla in each complex tibial organ of *H. pallitarsis* were generally lower than those found in *Hemideina*. The *H. pallitarsis* SGO had the most sensilla (~50), but still fewer than the *Hemideina* SGO (~70)^25^. For the IO, numbers of sensilla were similar in both genera, but *H. pallitarsis* had no separation into proximal and distal groups as found in *Hemideina* ^28^ and other ensiferans (tettigoniids^36–39^, cave cickets^24, 40^). In both genera the SGO sensilla were arranged in similar fashion.

In contrast, marked differences were seen in CA/CAH sensillum numbers and arrangement between the two genera. In *Hemideina*, the foreleg CA had about 400% more sensilla (~53) than observed in the *H. pallitarsis* foreleg CAH (~12); and the other two pairs of legs in *Hemideina* had about 300% more sensilla (~32, ~32, resp.) than those found in *H. pallitarsis* mid- and hind legs (~13, ~12 resp.)^28^. The high number of foreleg sensilla in *Hemideina* is associated with the development of tympanal membranes, whereas the similar sensillum numbers between thoracic leg pairs in *H. pallitarsis* are associated with the lack of such tympana. Furthermore, the distal sensilla in the CA of *Hemideina* form a double row of somata (with some triplets)^28^, also reported for the CA of several tettigoniid species^39, 41^. Such a double row of somata was not found in the atympanate CAH of *H. pallitarsis*.

The detailed differences in neuroanatomical arrangement of sensory elements in tympanate and atympanate weta, together with morphological differences in internal anatomy, suggest adaptations to detect different sensory modalities. In *Hemideina*, tibial adaptations for sound detection include two tympana on each foreleg, enlarged and flattened tympanal vesicles and large air chambers, derived from the anterior tracheae, which act to load the tympanum. This, in turn, oscillates the CA/IO mass against its dorso-posterior suspension points^20, 25^. This morphology is associated with a large number of serially graded sensilla in the CA, as also found in other tympanate ensiferans (tettigoniids^26, 36, 38^ and crickets^42, 43^). In contrast to such elaborately modified internal anatomy, *H. pallitarsis* lacked external tympana and showed a simpler internal anatomy. Furthermore, the small size of both tibial tracheae in *H. pallitarsis* suggests a high resistance to airborne sound energy transmission. The unspecialized foreleg anatomy, together with the evenly distributed IO and CAH sensillum numbers in all leg pairs, argues against airborne sound detection by the complex tibial organ of *H. pallitarsis*, and, instead, suggests that the IO/CAH performs a similar sensory function in all leg pairs, as discussed below. These anatomical features are compared between ground weta and tree weta in Table 2. In sum, the neuronal sensilla follow similar ground patterns in tympanate and atympanate organs, but with more CA sensilla associated with far-field tympanal hearing.

**Table 2:** Functional morphology of the complex tibial organ in tympanate tree weta (*Hemideina*) and atympanate ground weta (*Hemiandrus*).

	*Hemideina species* Tree weta	*Hemiandrus pallitarsis* Ground weta
Tympana	Large foreleg tympana	no tympanal membranes
Acoustic vesicles	Tracheal vesicles in tibia	no tracheal vesicles in tibia
SGO sensilla	Many SGO sensilla (~70)	medium number of SGO sensilla (~50)
CA sensilla foreleg	High number of CA sensilla (50–53)	rather low number of CAH sensilla (12)
Serial CA/CAH gradient	clear gradient in CA sensilla from T1 (high) to T3 (low)	no serial gradient in CAH sensilla
Size of CA/CAH somata	become smaller in distal CA	similar over CAH
Distal CA/CAH sensilla	double row of distal CA somata	no double row of CAH somata
IO anatomy	IO compact	prominent IO extends dorsally in tibia
IO mass	IO mass attached dorsally by connective strands	IO/CAH attached jointly at dorsal cuticle over broad area

### Functional morphology and attachment of the sensory organs in relation to vibration detection

The shapes and attachments of the distinct scolopidial organs determine their physiological activation. The SGO in *Hemiandrus* is a flat oval structure which occludes the dorsal hemolymph channel in the tibia. This morphology differs from the *Hemideina* SGO, which is pillow-shaped and hence may present an inertial mass that could resonate to vibratory stimuli^28^. In *H. pallitarsis*, the following morphological details give insight into the mechanism of action: a) the wafer-like mass of the SGO is thick around the semicircular posterior margin where it is solidly anchored to the posterior cuticlular wall, but becomes a thin membrane anteriorly; b) the semi-circular anterior margin gives rise to many thin strands attached to the anterior cuticular wall, and which emanate from parallel strap-like cells covering the distal SGO surface (Fig. 7c,e). Similar strands have been described for the SGO mass in Mantophasmatodea where they were interpreted as scolopidia^44^. Assuming the thin strands in *H. pallitarsis* are elastic, as indicated by their bent dorso-ventral form (Fig. 7f), the structure suggests that the SGO functions as a posteriorly restrained hinged-plate mechanism. Longitudinal compression waves travelling along the hemolymph channel could displace the more compliant anterior half of the SGO while the posterior part remains anchored to the posterior wall. This model is supported by the location of many SGO scolopidia along the anterior and dorsal margin of the SGO mass which appear to lie in the region of maximum deformation.

The SGO mass is well positioned to derive maximum stimulation within the dorsal hemolymph channel. It almost occludes the whole channel, thus exposing a large surface area to undergo hydrostatic displacement by oscillations, travelling proximally in the hemolymph channel, from substrate vibrations acting on the tarsal pads. Yet, the normal proximal movement of hemolymph in the dorsal channel^45^ would be uninterrupted because the hemolymph could flow through the mesh created by the marginal anterior strands. Furthermore, the dorsal hemolymph channel is wide distal to the SGO, but narrows considerably proximal to the SGO, as in *Hemideina*. This funneling effect could amplify compression waves in the hemolymph moving against the SGO and enhance its sensitivity^20^. It is not known whether any sensory noise in the SGO response is due to changing hemolymph pressure during normal circulatory function.

We show, for the first time, a differently specialised shape and location of the IO/CAH mass, compared to that found in *Hemideina*, and ensiferans in general. In *H. pallitarsis*, the sensilla of both organs (IO and CAH) are embedded in a single flattened triangular mass broadly attached along its entire vertical length to the anterior cuticular wall. In *Hemideina* the IO/CA mass is suspended in the hemolymph by two attachment strands and rests upon the inflated anterior tympanal vesicle, which is driven by tympanal movement. While the mass in *Hemideina* is attached to the modified tracheal system, the mass in *H. pallitarsis* is attached to the cuticular wall of the tibia. Both organisations differ from the tent-shaped IO septum^46^ and the thin tectorial membrane covering the CA in for-, mid- and hindlegs of tympanate tettigoniids^36, 38^. In tettigoniids^36, 38^ and cave crickets^40^, the IO is also proximally attached to the anterior inner tibia but the sensilla locate more medially^37, 38^ and the attachment is thus less close. With respect to function, the groups of IO and CAH sensilla in *H. pallitarsis* lie in two different planes (IO close to the cuticle, CAH more medial, Fig. 7ai,aiii), but they are embedded in a single mass attached to the inner cuticle on the anterior side of the tibia. It is likely that the IO and CAH sensilla function as a single sensory unit which responds to vibrations of the tibial cuticular wall, resonating in response to cuticle oscillations. This combined attachment would represent a unique cuticular vibration sensory organ common to all three thoracic leg pairs. Thus it likely provides a vibrational input pathway, via the cuticular surface of the leg, separate from that of the SGO.

Importantly, in *H. pallitarsis* the connection of the IO/CAH mass to the tibial tracheae is limited, resulting in almost complete mechanical isolation of the two sensory structures from the tracheal system. This is in contrast to the anatomically well-studied tettigoniids^31, 32, 36, 37, 47^ and cave crickets^40^, where the IO is located dorsally close to the anterior trachea and linked to the posterior trachea by tissue strands. In *H. pallitarsis*, the IO/CAH mass is prominently attached to the inside cuticle of the tibia and the CAH sensilla are not stabilised by dorsal supporting bands as found in the tettigoniid mid- and hindlegs^26, 38^. The above *H. pallitarsis* organisation is also very different from the location of the *Hemideina* IO/CA mass, which is attached dorsally onto the anterior trachea.

In summary, the atympanate condition in *H. pallitarsis* is accompanied by lack of adaptation for sound reception in the complex tibial organs of all three leg pairs (Table 2). Nevertheless, atympanate chordotonal organs may show some sound sensitivity, typically with high thresholds to low frequency stimuli^36, 40, 48–52^, due to induced vibration from high intensity (non-physiological) sound stimuli^48^. It is possible that this could also occur in *H. pallitarsis*. But since the IO/CAH mass lies dorsal and lateral to the tracheae, the indirect connective tissue link would present a high impedance, and an ineffective acoustic pathway.

### Implications for vibration detection and differentiation of several sensory organs

The organ morphology shows specialisations for detecting vibrations though fluid and cuticular pathways in the leg. This is considered adaptive since intraspecific communication for mate localization through substrate drumming appears not to be based upon auditory signal reception^12^. In *H. pallitarsis*, the different attachments of the SGO and the IO/CAH suggests different input pathways to these sensory organs: to the SGO via movements of the hemolymph and to the IO/CAH via the cuticle, respectively. The vibration sensitivity of the IO and CAH is presumably tuned to a broad frequency range. In other ensiferans, IO sensilla show the highest sensitivity to vibrations of 700–1000 Hz, however the tuning curves of individual sensilla generally tend to overlap and show no clear range fractionation^48, 53^. This observation is compatible with the functional morphology described above, in which the single IO/CAH mass presents a homogeneous mass that likely precludes frequency discrimination. Unfortunately, the spectral characteristics of the drumming signals have not been documented for *H. pallitarsis*. A possible tuning of the sensory organs to the conspecific vibrational signals remains to be investigated, in particular in relation to a role of plants in signal transmission by damping or filtering^54^. In *H. pallitarsis*, our preliminary recordings from sensory nerve 5B1 proved that the complex tibial organ to responds to sinusoidal vibrations of broad frequencies delivered to the tarsal pads, but distinct roles of specific scolopidial organs and their frequency tuning requires more detailed electrophysiological analysis.

### Comparative and evolutionary aspects: diversity of complex tibial organs in weta

For Ensifera in general, it is not clearly established in which lineage(s) tympanal hearing organs evolved nor where it was possibly lost secondarily^55^. The phylogeny of weta and Anostostomatidae is so far not well established, and in the phylogenetic relationships, atympanate *Hemiandrus* may not to be the most basal lineage^56, 57^. It may even be a paraphyletic group^57^. Likely, the most basal lineage of Anostostomatidae is the Australian genus *Transaevum* ^57, 58^. Remarkably, in *Transaevum laudatum* a single antero-dorsal tympanum has been described in the foreleg tibia^59^. Similar single foreleg tympana are present in some Australian Anostostomatidae (some species of *Hemiandrus* and an undescribed anostostomatine genus)^58^. While the Anostostomatidae of both Australia and New Zealand have Gondwana origins, during the prolonged geographical separation a great diversity of tympanal structures developed: large paired tympanal membranes associated with hearing organs are found in New Zealand tree weta, giant weta and tusked weta, single tibial tympana are found in Australian anostomatids including species from the genus *Hemiandrus*, while the New Zealand ground weta, as well as other Australian anostostomatid groups, lack obvious tympana^7, 8, 58–60^. Thus, tympanal membranes can be present or absent in different anostostomatid subfamilies (Supplementary Table 1).

Broader investigations on these diverse sensory structures are needed to delineate their sensory functions and evolutionary diversification, to combine both neuroanatomical and phylogenetic aspects. So far, the hearing systems with single tympana have not been characterised further with respect to either functional morphology or sensory physiology (e.g. hearing thresholds). Yet, the conserved occurrence of the complex tibial organ with four sensory organs in Tettigonioidea including Anostostomatidae strongly suggests that these species also have auditory systems with a CA or its atympanate homologue^61^. The ancestral sensory function of the CAH is also not clear, but we show here for the ground weta, as well as previously for other atympanate Ensifera, that they have a fully developed complex tibial organ with a CAH homologous to auditory sensilla of tympanate tree weta and tettigoniids^61–65^. The CAH is possibly a detector for vibrational stimuli in higher frequency ranges^63^. For the *Hemiandrus* IO/CAH, the broad attachment to the cuticle as an input pathway, the isolation from the tracheal system, and the similar neuronal structure in all leg pairs favour a function of vibration detection, shared in all leg pairs. This implies that the complex tibial organs in the tettigonoid leg have provided a base plan for the evolution of different structural and functional sensory systems through divergent morphological elaboration of shared neuronal, tracheal and membranous elements. The New Zealand and Australian anastostomatid insects thus provide a rich potential for studying the evolution and diversification of adaptations in mechanosensory systems and associated signalling behaviours.

## Electronic supplementary material

Distribution of tympanal membranes in species of different groups of Anostomatidae

Supplementary Dataset 1
