# Supplementary material for: The complex tibial organ of the New Zealand ground weta: sensory adaptations for vibrational signal detection
Source: Sci Rep. 2017 May 17;7:2031. doi: 10.1038/s41598-017-02132-1 (PMC5435688; doi:10.1038/s41598-017-02132-1)
Supplement: Supplementary file 1 — Distribution of tympanal membranes in species of different groups of Anostomatidae [file 41598_2017_2132_MOESM1_ESM.doc]

Supplementary table to:

Johannes Strauß, Kathryn Lomas, Laurence H. Field

**The complex tibial organ of the New Zealand ground weta: sensory adaptations for vibrational signal detection.**

**Supplementary Table 1**: Distribution of tympanal membranes in species of different groups of Anostomatidae^1, 2, 3^.

| **Taxon** | **Tympana present** | **Tympana absent** | **Both** |
| --- | --- | --- | --- |
| Anabropsinae |  |  | x |
| Cratomelinae |  | x |  |
| Lutosinae | x |  |  |
| Anostomatinae |  |  | x |
| Leilomelinae |  | x |  |
| Deinacridinae |  |  | x |

**References**

^1^Johns, P. M. The Gondwanaland Weta: Family Anostostomatidae (formerly in

Stenopelmatidae, Henicidae or Mimnermidae): nomenclature problems, world checklist, new genera and species. *J Orthopt Res* **6**, 125-138 (1997)

^2^Gorochov, A. V. The higher classification, phylogeny and evolution of the superfamily Stenopelmatoidea in *The Biology of Wetas, King Crickets and their Allies* (ed Field, L.) 3-33 (CABI Publishing, 2001)

^3^Monteith, G. & Field, L. Australian King Crickets: Distribution, habitats and biology

(Orthoptera: Anostostomatidae) in *The Biology of Wetas, King Crickets, and their Allies* (ed Field, L.) 79-94 (CABI Publishing, 2001)
